# Study on the preparation process of quinoa anti-hypertensive peptide and its stability

**DOI:** 10.3389/fnut.2022.1119042

**Published:** 2023-01-18

**Authors:** Xing Fan, Xuemei Ma, Ruxianguli Maimaitiyiming, Aihemaitijiang Aihaiti, Jiangyong Yang, Xianai Li, Xiaoyun Wang, Guangxian Pang, Xiaolu Liu, Chenggong Qiu, Redili Abra, Liang Wang

**Affiliations:** ^1^College of Life Science and Technology, Xinjiang University, Urumqi, China; ^2^Xinjiang Arman Food Group Co. Ltd., Urumqi, China; ^3^Shenxin Science and Technology Cooperation Base Co. Ltd., Urumqi, China

**Keywords:** quinoa, fermentation, peptides, amino acids, molecular weight, stability

## Abstract

Quinoa seeds are a food resource rich in protein, vitamins, minerals, and other functional components such as polyphenols, polysaccharides, and saponins. The seeds have become favored by modern consumers due to being gluten-free and featuring a high protein content. This study focused on the preparation of quinoa peptides by short-time enzymatic-assisted fermentation. Quinoa flour (QF) was mixed with water in a certain ratio before being enzymatically digested with 0.5% amylase and 0.1% lipase for 6 h. Then, 16 bacterial taxa were used for fermentation, respectively. The peptide content in the resulting fermentation broths were determined by the biuret method. The dominant taxon was then identified and the peptide content, amino acid distribution, and molecular weight distribution of the prepared quinoa peptides were analyzed. Further, the temperature, pH, metal ions, organic solvents, ion concentration, and anti-enzyme stability of the quinoa anti-hypertensive peptides of different molecular weights after fermentation with the dominant taxon were investigated. Finally, the inhibitory activity of fermented quinoa peptides on bacteria was studied. The results show that the peptide content of the fermentation broth reached 58.72 ± 1.3% at 40 h of fermentation with *Lactobacillus paracasei* and the molecular weights of the hydrolyzed quinoa peptides were mainly distributed below 2 kDa by polyacrylamide gel. The Angiotensin Converting Enzyme (ACE) inhibition and peptide retention of the 0–3 kDa quinoa peptides were screened to be high and stable. At the same time, the inhibitory activity of quinoa peptide after fermentation on E. coli was obvious. This study provides a theoretical basis for further research on quinoa peptide and its application in industrial production, and also lays a foundation for the later application of polypeptides in new food and chemical products.

## 1. Introduction

Quinoa, native to the Andes of South America (1), is a dicotyledonous plant of the Amaranthaceae family. Its seeds are particularly rich in protein and contain a higher proportion of the macronutrient compared to other popular crops, such as rice and corn. The amino acid composition of quinoa resembles that of the “ideal protein” recommended by the Food and Agriculture Organization of the United Nations (FAO) (2). Extracts of quinoa protein have been shown to exhibit a range of biological activities. In addition to containing high amounts of protein (2), lipids (3), fiber, vitamins (4), and minerals (5), the protein within quinoa can also be extracted for its use as a supplemental food additive. Quinoa protein features a relatively good amino acid balance and its amino acid profile resembles that of bovine casein (6), rendering it a high-quality plant-based source of protein (7). Quinoa protein possesses a range of biological activities, such as antioxidant (8), hypoglycemic (9), immunomodulatory, hypolipidemic (7), and prebiotic activities. The high content of free tryptophan in quinoa can enter the brain and provide a raw material for the synthesis of neurotransmitters for the central nervous system (10). The protein content of quinoa ranges from 9.1 to 15.7% (6) depending on the variety, and the nutritional value and protein content of quinoa seeds are higher compared to that of barley, wheat, and millet (6, 11). The main component of quinoa seeds is starch (12), and during proteolysis, the rate of its decomposition is influenced by the starch properties. Therefore, in this experiment, we first hydrolyzed quinoa flour (QF) by enzymatic hydrolysis and then fermented the resulting enzymatic hydrolysate.

In recent years, most studies conducted on the preparation of quinoa protein (13) have employed the alkali-acid precipitation method, which is the most used method in the food-processing industry, owing to its methodological simplicity, low associated cost, and capacity for large-scale production. However, pH adjustment during the process requires the use of large amounts of acids and bases, thereby causing potential contamination and denaturation of the proteins. The enzymatic preparation of proteins has attracted some favor due to involving only mild production conditions, featuring rapidness, and having a simple operation process, however, the method often results in a bitter taste of the final product. The fermentation process is widely employed due to the health-promoting effects conferred by peptides prepared by microbial fermentation, the associated alleviation or prevention of various health conditions, and the positive effects that the process contributes by improving the functional properties of the raw materials in tandem with reducing anti-nutritional factors (14–16). Fermentation has been widely used in the food industry for the processing of various nutritional products, including soybean meal peptides (17), fish dew peptides (18), corn gluten meal peptides (19), and canola peptides (20). Being particularly suited for the preparation of small molecule peptides, the fermentation method has the advantages of being economical, producing no pollution, and being suitable for industrial production. However, the process of fermentation is readily altered by the species of microorganisms used and the fermentation conditions employed. Zhao et al. (21) improved the defatting rate in soybean meal by coupling enzymatic digestion with fermentation, and this technique serves as the basis for our study.

ACE is a blood pressure-related protease involved in the ACE-renin-angiotensin system (RAS) that exerts impacts on human health (22). In recent years, bioactive peptides with ACE inhibitor activity have been shown to be released from various naturally derived proteins, and hence have great potential as alternatives to ACE inhibitors due to the possibility of lacking the range of side effects associated with traditional ACE inhibitor drugs (23). Peptides extracted from whey protein hydrolysate (24), mushrooms (25), corn (26), and Qingke Baijiu (27) have been reported to exert inhibitory effects on ACE. However, most of these studies only investigated the structural identification and inhibition of these peptides, while there have been fewer studies to assess the stability of the peptides. It is worth noting that bioactive peptides must undergo gastrointestinal digestion to reach the target site in order to exert their biological effects in the human body (28). Furthermore, various peptide treatment steps and conditions may alter the bioactive substances; these include enzymaticlysis, fermentation, drying, transportation, processing, pH, temperature, metal ions, organic solvents, light, and ionic intensity. The use of bioactive peptides as active ingredients is premised on the fact that they can remain stable in different environmental systems and across different processing methods (29). The microbiological contamination is a major cause of food shelf life and also has the potential to affect the health of consumers. In industry, preservatives containing chemicals are commonly used to retard the growth of microorganisms or to avoid spoilage caused by chemical changes. Bacteriostatic peptides can replace chemical preservatives as natural preservatives for food preservation. Therefore, this study investigated the stability, preparation process, and antibacterial activity of quinoa peptides under different environmental conditions. The results of this study are beneficial for the application of quinoa peptides in novel food and chemical products.

The purpose of this study was to find the taxon most suitable for quinoa peptide fermentation through a single-factor test and to obtain the best fermentation process for their preparation. The stability of the isolated peptides to heat, pH, metal ions, organic solvents, ionic strength, light, and simulated gastrointestinal digestion treatments was then further investigated. Our results provide insight useful for the development of new natural ACE inhibitors as well as the high-value utilization of plant-derived proteins.

## 2. Materials and methods

### 2.1. Materials and instruments

Quinoa was from Xinjiang, China. All solvents and chemicals were of analytical reagent grade or higher. Equipment included a water bath, spectrophotometer, centrifuge, incubator, electrophoresis tank, microscope, swirl mixer, and 80 mesh sieve. The amylase activity was 3,700 U/g and the lipase activity was 100,000 U/g. The equipment and conditions used for high-performance liquid chromatography were as follows: Agilent 1100 HPLC system (Agilent, USA), including online degassing device (G1322A), quadruple pump (G1311A), autosampler (G1313A), VWD detector (G1314A), and 1525 Binary HPLC Pump (Waters Co.); methanol (chromatographic pure) and acetonitrile (chromatographic pure) were purchased from TEDIA (United States); tetrahydrofuran, triethylamine, hydrochloric acid, crystalline tetrahydrofuran, triethylamine, hydrochloric acid, and sodium acetate trichloroacetic acid were all of analytical grade in purity; water was Millipore ultrapure water. Seventeen kinds of amino acid standards (Aspartic acid, Histidine, Glutamic acid, serine, Glycine, Threonine, Alanine, Arginine, Tyrosine, Cystine, Valine, Methionine, Phenylalanine, Isoleucine, Leucine, Lysine, and Proline), OPA, FMOC, and angiotensin-converting enzyme (ACE) were purchased from Sigma (United States). FAPGG was purchased from Solarbio (China). S. aureus and E.coli was purchased from Bainer Biotech (China).

### 2.2. Extraction of quinoa protein

**Figure 1:**
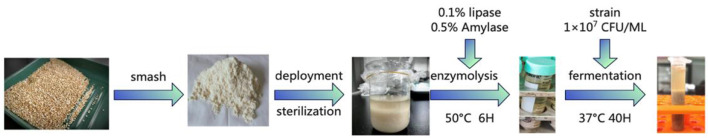
Flow diagram of quinoa peptide processing.

The quinoa seeds were **first** pulverized with a grinder and passed through an 80-mesh sieve. The resulting powder was then suspended in water (1:15, w/v) for mixing, heated in a water bath at 85°C for 30 min to reduce endogenous microbiota, and left to reach room temperature. Lipase (0.1% w/w to QF) and amylase (0.5% w/w to QF) were added to the room temperature (25°C) quinoa liquor to initiate the enzymatic reaction. After a 6 h bath at 50°C, heat at 85°C for 30 min to terminate the reaction (30). After inactivation, the quinoa solution was brought to room temperature and split such that 16 single bacteria were inoculated into respective solutions, each at a density of 1 × 10^7^ CFU/ml (31). Flow diagram of quinoa peptide processing in Figure 1. The peptide content was measured every 8 h for a total of 48 h in a 37°C incubator. After the fermentation was complete, the fermentation broth was separated using ultrafiltration centrifuge tubes and stored at 4°C. The 16 bacterial taxa were: *Bacillus subtilis, Lactococcus lactis subsp, Kluyveromyces marxianus, Lactobacillus helveticus, Bifidobacterium, Bacillus natto, Lactobacillus rhamnosus, Lactobacillus thermophilus, Lactobacillus acidophilus, Lactobacillus casei, Lactobacillus paracasei, Lactobacillus plantarum, Lactobacillus fermentum, Lactobacillus bulgaricus, Lactobacillus reuteri, and Lactobacillus pentosus*.

### 2.3. Determination of peptide content

The peptide content was modified by the method reported by Cotton et al. (32). A 1 ml volume of sample solution was added to 1 ml of 15% trichloroacetic acid aqueous solution, mixed, and allowed to stand for 10 min, then centrifuged at 4,000 r/min for 10 min. A 1 ml volume of supernatant was then taken, mixed with Biuret reagent, and allowed to stand for 30 min. At the same time, 1 and 4 ml of Biuret reagent were mixed to serve as the blank and the absorbance was measured at 540 nm (33). The peptide concentration C (mg/ml) in the hydrolysis product was calculated from the standard regression equation.

### 2.4. Kjeldahl nitrogen determination

After determining the optimal fermentation time for each taxon, the fermentation broth was dried and the nitrogen content was detected according to the Kjeldahl method (GB5009.5-2016).

Weighed 2–5 g of semi-solid specimen into a digestion tube and added 0.4 g of copper sulfate, 6 g of potassium sulfate, and 20 ml of sulfuric acid to the digestion oven. When the digestion temperature reached 420°C, the digestion was continued for a further 1 h. The liquid in the digestion tube became observably green and transparent by the time it was removed and cooled. Then, 50 ml of water was added and the Digestive juices was automatically added, distilled, and titrated before the titration data were recorded on an automatic Kjeldahl nitrogen tester (with sodium hydroxide solution, hydrochloric acid standard solution, and boric acid solution containing mixed indicator A or B).

Dried the peptide solution obtained above during the assay to obtain a semi-solid specimen. The hydrolysis of quinoa fermented by different taxa was calculated to have undergone a water loss of 85% of the fermentation residue and to have had a protein conversion factor of 6.25.

### 2.5. SDS-PAGE

SDS-PAGE was carried out according to the procedure outlined by Laemmli (34).

#### 2.5.1. Sample processing

The sample was denatured before loading. The sample was mixed with the loading buffer and placed in an EP tube, heated at 95°C for 5 min, and immediately either placed on ice or stored at 20°C for further analysis.

#### 2.5.2. Sample loading

The separating and stacking gels were prepared according to the Table 1. Approximately 20 μl of each sample was transferred into each well for electrophoresis.

**Table 1:** Gel preparation.

**Chemicals**	**Separation gel**	**Stacking gel**
Selation scheme	15%	5%
30% Acr/Bis (29:1)	5 ml	0.83 ml
1.5M Tris-HCl buffer (PH 8.8)	2.5 ml	/
ddH20	2.3 ml	3.42 ml
10% SDS	0.1 ml	0.05 ml
10%PAGE Glue coagulant	0.1 ml	75 μL
PAGE Gel accelerator	0.01 ml	7.5 μL
1M Tris-HCl buffer (PH 6.8)	/	0.625 ml

#### 2.5.3. Running glue

Electrophoresis was carried out by the warm flow method, as follows: the current was kept constant at 20 mA, the voltage was 100 V, and the running time was 3–4 h. The electrophoresis apparatus was ice-bathed throughout the process.

#### 2.5.4. Staining and decolorization

Staining was performed using Coomassie Brilliant Blue R-250 and decolorization was performed with decolorization solution.

### 2.6. Determination of the molecular weight distribution

(1) The main operating parameters of the instrument were as follows. Column: TSKgel 2000 SWXL 300 mm x 7.8 mm; mobile phase: acetonitrile/water/trifluoroacetic acid, 45/55/0.1 (V/V); detection: UV 220 nm; flow rate: 0.5 ml/min; column temperature: 30°C.

(2) Sample preparation: 100 mg of each sample was taken in 10 ml volumetric bottles, diluted with mobile phase to scale, and filtered with 0.45 μm microfilm for sample supply.

(3) The sample solution was analyzed under the above chromatography conditions and the data were processed with GPC software to obtain the phase of the peptide in the sample, the pair of molecular mass distribution, and its distribution range.

### 2.7. Amino acid analysis

The amino acid composition was determined by acid hydrolysis, derivatization, and HPLC quantification according to the method described by Ponka et al. (35). The results are expressed in mg per 1 ml of dry matter.

Mobile phase A (pH = 7.2): 27.6 mmol/L sodium acetate-triethylamine-tetrahydrofuran (500:0.11:2.5, v/v).

Mobile phase B (pH = 7.2): 80.9 mmol/L sodium acetate-methanol-acetonitrile (1:2:2, v/v).

An Agilent Hypersil ODS column was used (5 μL, 4.0 mm × 250 mm); the gradient elution used the following elution program: 0 min, 8% B; 17 min, 50% B; 20.1 min, 100% B; 24 min, 0% B; the mobile phase flow rate was 1.0 ml/min; the column temperature was 40; UV detector (VWD). The wavelength was 338 nm and proline was detected at 262 nm; the amino acid content was quantified by the external standard method.

### 2.8. Isolation of peptides from QF

The fermentation broth was ultrafiltered using ultrafiltration centrifuge tubes with cut-off values of 3 and 10 kDa to obtain peptides of 0–3 kDa (QPH-2) and 3–10 kDa (QPH-1), which were then freeze-dried and stored at −80°C until use.

### 2.9. *In vitro* assay for ACE inhibition rate

In a 96-well plate, 40 μL of sample solution, 50 μL of FAPGG, and 10 μL of the ACE enzyme were added to the sample wells; 40 μL of HEPES buffer solution, 50 μL of FAPGG, and 10 μL of the ACE enzyme were added to the blank wells. The absorbance values were measured at 340 nm and recorded using an enzyme marker, then measured and recorded again after 30 min at 37°C. The following equation was used to determine the degree of ACE inhibition:


R= [1−(a1− a2)] / (b1− b2) ]× 100

where:

R, ACE inhibition, %

a1, initial absorbance of blank well at 340 nm

a2, absorbance of blank well at 340 nm after 30 min at 37°C

b1, initial absorbance of sample well at 340 nm

b2, initial absorbance of sample well at 340 nm.

### 2.10. Stability studies

#### 2.10.1. Measurement of thermal stability

Peptide solutions of different molecular weights were prepared at a concentration of 2 mg/ml and treated in a water bath at 60, 70, 80, 90, and 100°C, respectively, for 2 h (36). The samples were then cooled rapidly to room temperature in an ice-water bath for the determination of the peptide content and ACE inhibitory activity.

#### 2.10.2. Measurement of pH stability

The peptide samples were dissolved in Na_2_HPO^4−^citrate buffer (0.1 mol/L) at pH 2, 3, 4, 5, 6, 7, 8, 9, and 10, respectively, and prepared to a mass concentration of 200 μg/ml (28). The pH was adjusted with HCl and NaOH (1 mol/L), respectively, and the peptide content and ACE inhibitory activity were then determined by standing for 2 h at room temperature.

#### 2.10.3. Measuring the stability of metal ions

The peptide solution was prepared at a concentration of 2 mg/ml and metal salts containing K^+^, Ca^2+^, Zn^2+^, and Ba^2+^ were added to the solution, respectively, to achieve a mass concentration of 250 μg/ml (37). The peptide content and ACE inhibitory activity were finally determined by standing for 2 h at room temperature.

#### 2.10.4. Measuring *in vitro* simulated digestive stability

##### 2.10.4.1. Pepsin digestion test

The peptide solution was prepared at a concentration of 1 mg/mL and the pH of the system was adjusted to 2.0 with hydrochloric acid. Then, pepsin (4% of the mass of the quinoa peptides) was added, stirred well, and incubated in a water bath at 37°C for 2 h. Following incubation, the solution was immediately boiled in a water bath for 15 min and then cooled and centrifuged at 4,000 r/min for 15 min. The supernatant was collected, the pH was adjusted to 8.3 (38), and the peptide content and ACE inhibition activity were determined.

##### 2.10.4.2. Trypsin digestion test

The peptide was prepared at a concentration of 1 mg/mL and the pH of the system was adjusted to 7.5 with NaOH solution. Trypsin was added (the addition amount was 4% of the mass of the quinoa peptides), stirred well, and kept in a water bath at 37°C for 2 h. Following incubation, the solution was immediately boiled in a water bath to inactivate the enzyme for 15 min, then cooled and centrifuged at 4,000 r/min for 15 min. The pH was then adjusted to 8.3 and the peptide content and ACE inhibitory activity were measured.

#### 2.10.5. Measuring the stability of organic solvents

Peptide solutions of different molecular weights were prepared at a concentration of 2 mg/ml and added to methanol, ethanol, and propanetriol, respectively, each in a concentration gradient of 10, 20, 30, 40, and 50% before being mixed on a shaker for 2 h. The peptide content and ACE inhibitory activity were then determined.

#### 2.10.6. Measuring the stability of light

Peptide solutions of different molecular weights were prepared at a concentration of 2 mg/ml and placed at room temperature protected from light for 2, 4, 6, 8, and 10 h to determine the peptide content and ACE inhibitory activity.

#### 2.10.7. Stability of measured ionic strength

Peptide solution was prepared at a concentration of 2 mg/ml and added to NaCl solution to form a concentration gradient of 0.2, 0.4, 0.6, 0.8, and 1 mol/l, respectively. Each solution was centrifuged at 4,000 r/min for 15 min. The resulting supernatant was collected to determine the peptide content and ACE inhibition activity.

### 2.11. Test method for bacteriostatic activity

Antimicrobial activity was evaluated by turbidimetric analysis. Sample or positive control (100 μl) was mixed with 100 μl of bacterial suspension, and the resultant solution was incubated for 24 h at 37°C. A 96 well-microplate reader was used to measure the absorbance at 600 nm (39). sterile water was used as a negative control (NC). ABR denotes the antimicrobial rate of FCAP for E. coli or S. aureus, which can be calculated as follows:


ABR=(ODNC - ODsample)/ODNC×100%

where OD denotes the absorbance of the different solutions in 600 nm. NC denotes the negative control solution, and the sample denotes the test sample solution.

### 2.12. Statistical analysis

All results were expressed as the means ± standard deviations (S.D.). The differences were tested by one-way ANOVA followed by Duncan's multiple-range test. All statistical analyses were conducted using the SPSS software package (Version 25, SPSS Inc, Chicago, IL, USA) with a significance level of 0.05.

## 3. Results and discussion

### 3.1. Determination of the peptide content

**Figure 2:**
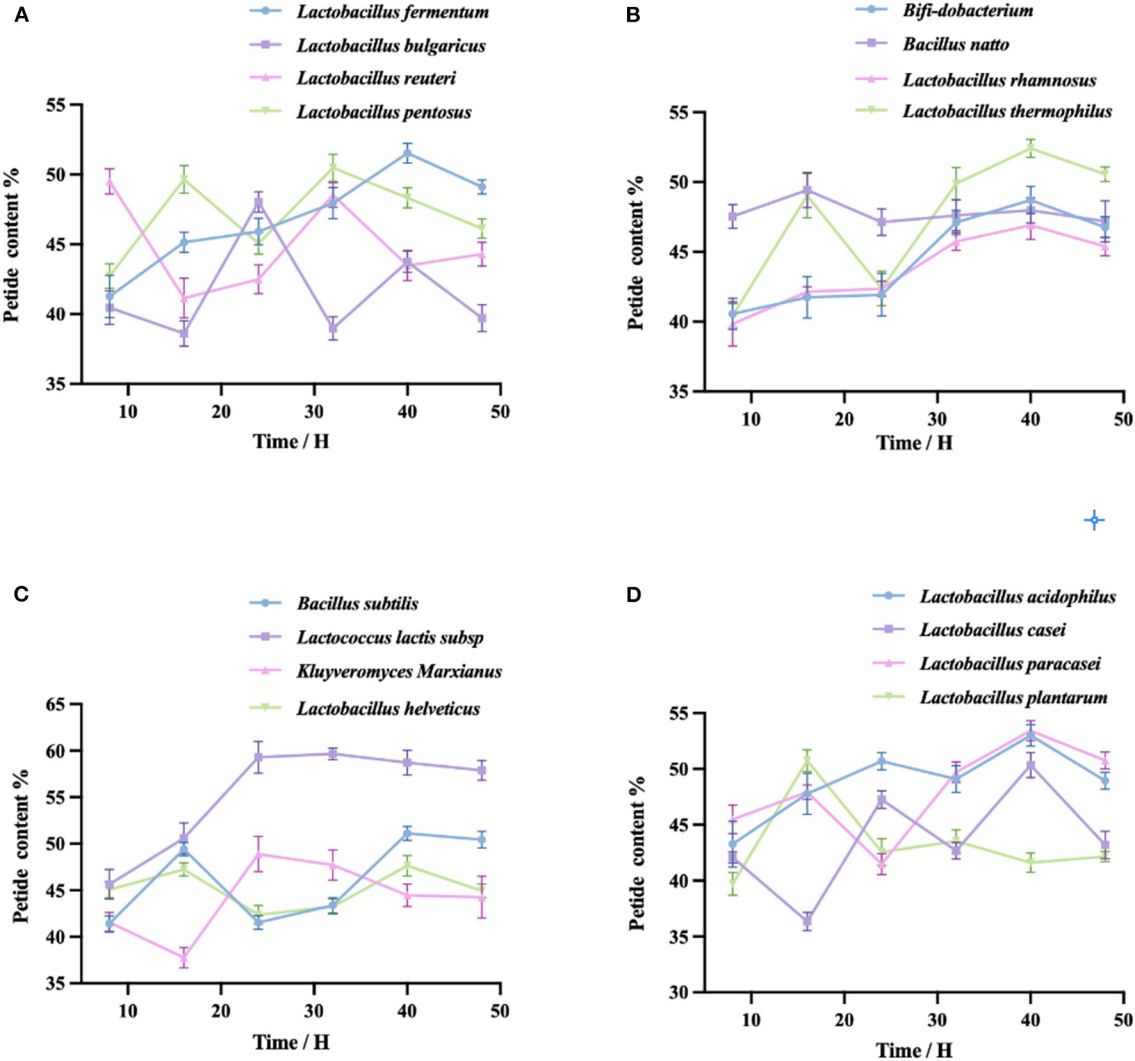
**(A–D)** Fermentation of QF with each of the 16 bacterial taxa. The peptide content was measured by the Biuret method. Variation in peptide yield (%) during the fermentation of quinoa by different taxa. Values are expressed as the mean ± standard deviation of three replicates.

As shown in the four figures, the peptide yield of fermented quinoa peaked at 40 h for most taxa and then either remained stable or decreased (except in the cases of *Lactobacillus reuteri* and *Lactobacillus plantarum*). The highest peptide yield was generated by *Lactococcus lactis subsp*. (58.72 ± 1.32%); the lowest was generated by *Lactobacillus plantarum* (41.62 ± 0.86%). The four taxa shown in Figure 2A exhibited different peptide yield trends throughout the process of fermentation. The peptides produced by *Lactobacillus reuteri* and *Lactobacillus bulgaricus* first decreased, then increased, then decreased once more; conversely, the peptides of *Lactobacillus pentoses* increased first, then decreased, then increased again; the peptide yield of *Lactobacillus fermentum* initially increased, then slowly decreased after reaching its peak value at 40 h. The four bacteria in Figure 2B, except for *Lactobacillus thermopiles* and the other three bacteria, exhibited a shared trend of fermentative peptide production: all yields gradually increased, then decreased slowly after reaching their peak values at 40 h. The polypeptide yield of *Lactobacillus thermopiles increased* first, then decreased, then increased, and then gradually decreased after reaching the highest peak. In Figure 2C, the polypeptide yield of *Lactococcus lactis subsp*. initially increased and then gradually decreased after peaking at 32 h. The polypeptide yields of *Bacillus subtilis* and *Lactobacillus helveticus* were the first to increase and then gradually decline after reaching their first peaks, then gradually further decreased after reaching their second peaks at 40 h. The peptide yield of *Kluyveromyces Marxianus* was first reduced and then gradually continued to decrease after reaching its peak at 24 h. In Figure 2D, the peptide yield of *Lactobacillus plantarum* first increased, then decreased after reaching its peak at 16 h. The peptide yield of *Lactobacillus acidophilus* continued to increase, gradually increased after having reached the lowest point at 32 h, and then gradually decreased after reaching the highest peak at 40 h. The peptide yields of *Lactobacillus casei* and *Lactobacillus paracasei* both fluctuated and reached their peak values at 40 h.

By detecting the peptide yields of the 16 taxa of bacteria, we found that the peptide yields of some taxa decreased within the first 8–16 h of fermentation. This may be attributable to the fact that the taxa were in the adaptation stage during this period, hence grew slowly, exhibited limited enzyme production, and produced lower peptide yields (40). With the extension of the fermentation time, the growth of the taxa gradually stabilized and the amount of enzyme production increased, thus increasing the peptide yields. When fermentation ended, due to the scarcity of nutrients remaining in the fermentation system and the large number of metabolic wastes, the dissolved oxygen would have been insufficient, which in turn would have affected the synthesis of enzymes. Under these conditions, enzyme production reaches saturation and the excessive hydrolysis of proteases leads to the production of peptides. This explanation is consistent with Yang's research results (41). By analyzing the data, we used 40 h as the fermentation endpoint and selected 8 single bacteria from 16 single taxa for further study.

### 3.2. Kjeldahl nitrogen determination

**Figure 3:**
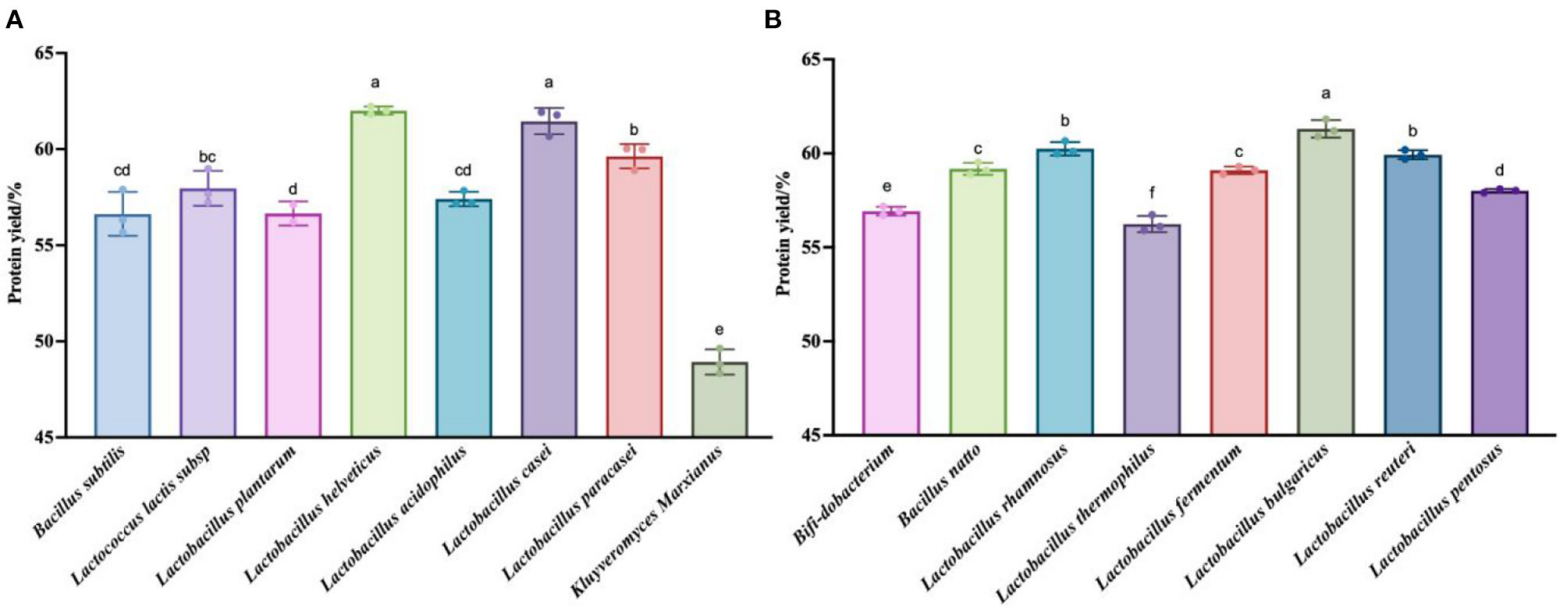
Detection results of the protein content in the fermentation residue. **(A)** Hydrolysis degree produced by fermentation of quinoa by 8 strains (*Bacillus subtilis, Lactococcus lactis subsp, Lactobacillus plantarum, Lactobacillus acidophilus, Lactobacillus casei, Kluyveromyces Marxianus*) at their respective optimal fermentation time. **(B)** Hydrolysis degree produced by fermentation of quinoa by 8 strains (*Bifi-dobacterium, Bacillus natto, Lactobacillus rhamnosus, Lactobacillus thermophilus, Lactobacillus fermentum, Lactobacillus bulgaricus, Lactobacillus reuteri, Lactobacillus pentosus*) at their respective optimal fermentation time. The values are presented as the mean of three replicates ± standard deviation. Dip-value ≤0.05.

To verify that the taxa were indeed effective at fermenting quinoa, we chose to measure the total nitrogen content of the fermentation residues obtained at the points representing the highest polypeptide content of each of the 16 taxa, then calculated the degree of hydrolysis of the quinoa protein. Figure 3 shows that the degree of fermentation broth hydrolysis exceeded 45% for all taxa. This method extracts quinoa protein from quinoa than the enzymatic hydrolysis method (black quinoa 21%, red quinoa 12.26%, gray quinoa 12.26%) (42) and the degree of hydrolysis (DH = 40.9%) of black soybean peptide prepared by microwave-assisted enzymatic hydrolysis (43) was high. The reason for this phenomenon may be due to any number of causes, including the different detection methods used to measure the degree of hydrolysis, the possibility of experimental error in the drying of the residue, or the fact that this method is indeed effective.

### 3.3. SDS-PAGE

**Figure 4:**
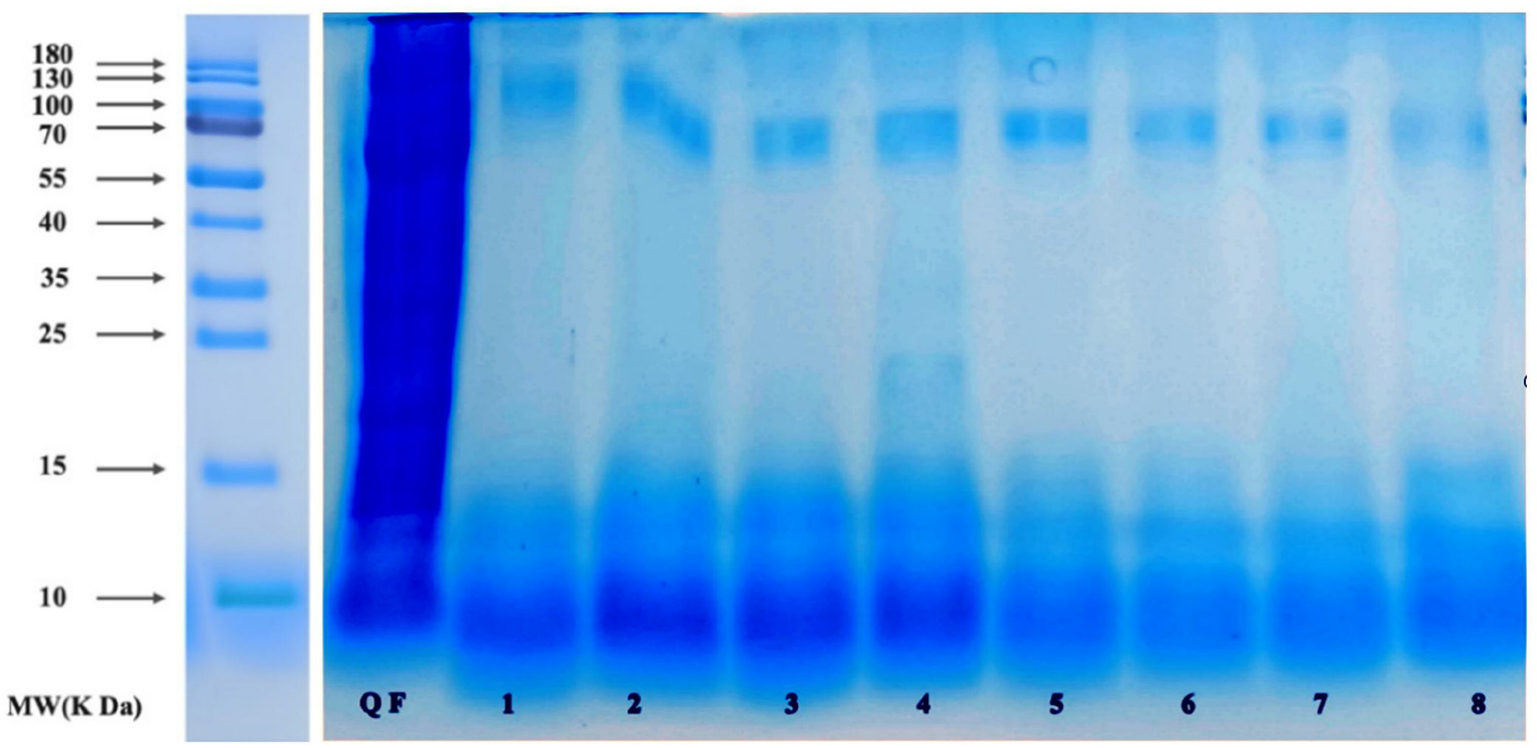
SDS-PAGE pattern of QF after fermentation. 1: QF fermented by *Bifi-dobacterium*. 2: QF fermented by *Lactobacillus casei*. 3: QF fermented by *Lactobacillus thermophilus*. 4: QF fermented by *Bacillus subtilis*. 5: QF fermented by *Lactobacillus fermentum*. 6: QF fermented by *Lactobacillus acidophilus*. 7: QF fermented by *Lactobacillus paracasei*. 8: QF fermented by *Lactococcus lactis subsp*.

The quinoa polypeptides produced after fermentation by the eight selected taxa (Figure 4) were analyzed by sodium dodecyl sulfate-polyacrylamide gel electrophoresis (SDS-PAGE). As can be seen from Figure 3, the molecular weights of the quinoa polypeptides obtained after fermentation were mostly concentrated below 10 kDa. This result was consistent with the results obtained by Shiva et al. for the hydrolysis of quinoa peptides with trypsin (8). We can see that the protein density among the raw materials was relatively high and the MW reached 180 kDa. The number of proteins in the fermentation broth decreased with increasing MW. The bands of quinoa polypeptides produced by bacterial taxa No. 1–4 were concentrated below 16 kDa, with some shallow bands at 25 kDa. The molecular weights of quinoa polypeptides produced by bacterial taxa No. 5–8 were concentrated at 11 kDa. Bacterial taxa No. 4 also generated a band at 12.5 kDa, but the protein density was low; after conducting an electrophoresis color comparison, we found that the bands produced by bacterial taxa No. 1–4 had higher protein densities within 10–15 kDa compared to those of bacterial taxa No. 5–8. In addition, these 8 bacteria each had a lower density band at 70 kDa. These bands may be enzymes with larger molecular weights that were added during the previous enzymatic hydrolysis process or they may be macromolecular proteins in the raw materials that were not decomposed during the fermentation process. In addition, considering the peptide yield shown in Figure 2, we selected the four taxa (*Lactococcus lactis subsp, Lactobacillus paracasei, Lactobacillus acidophilus, Lactobacillus fermentum*) to ferment quinoa peptides for further analysis.

### 3.4. Distribution of molecular weight

**Figure 5:**
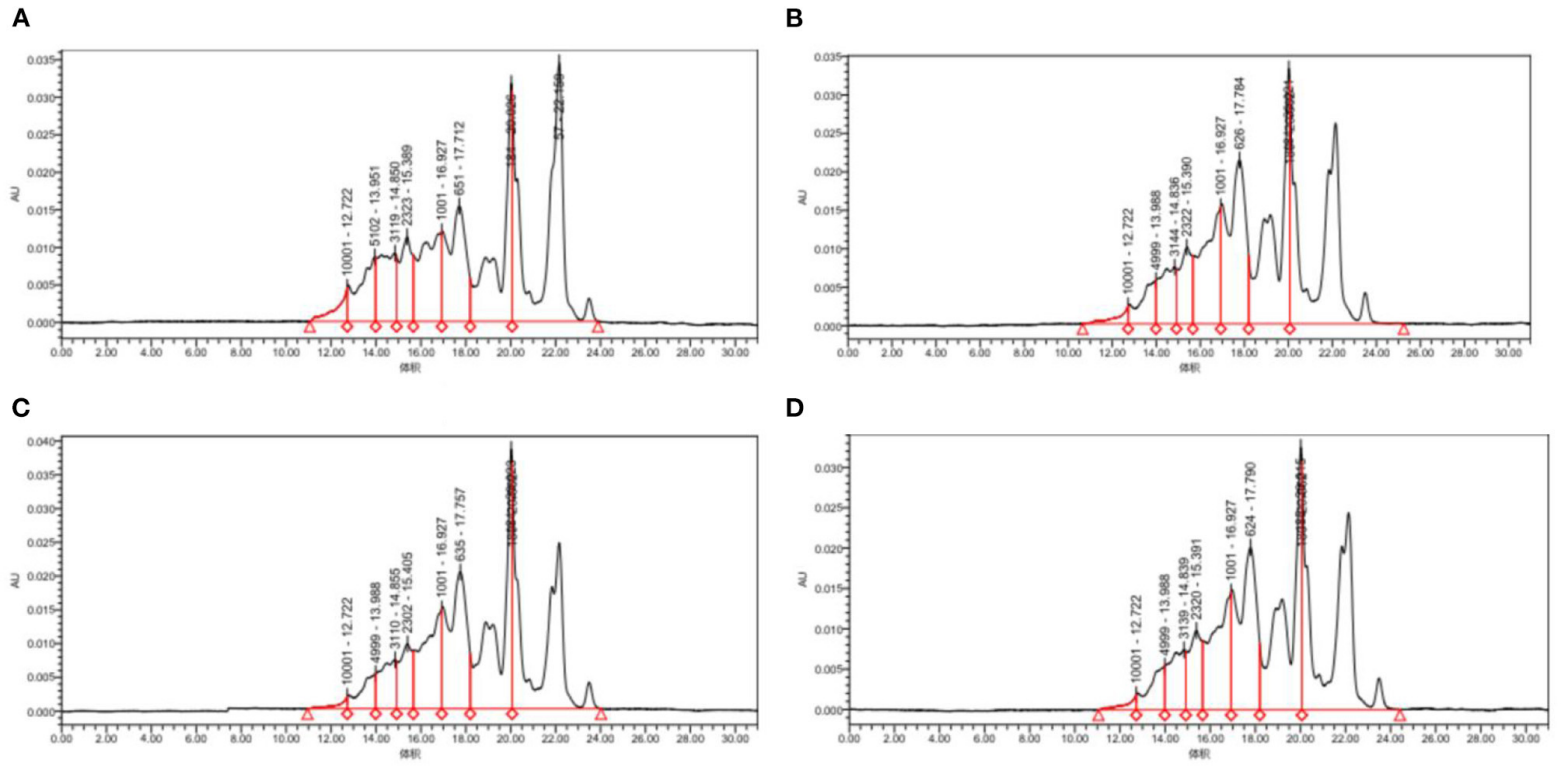
Molecular weight distribution of quinoa proteins hydrolyzed by different bacterial taxa. **(A)** QF fermented by *Lactococcus lactis subsp*. **(B)** QF fermented by *Lactobacillus paracasei*. **(C)** QF fermented by *Lactobacillus acidophilus*. **(D)** QF fermented by *Lactobacillus fermentum*.

Figure 5 and Table 2, the peak times of the four taxa were all in the range of 19–23 min, corresponding to the range of protein MW distributions below 2 kDa. According to the calculation of the peak area, the molecular weight distributions of proteins generated by the four bacteria were relatively similar, with most distributed in the range of 0–2 and 2–5 kDa. Among them, *Lactococcus Lactis subsp* was slightly different from the other three bacteria as its content of 0–2 kDa proteins was significantly lower (*p*<0.05), while the content of proteins with mass values of 2–5, 6–10, and 10 kDa and above was significantly higher than that produced by the other three bacteria (*p* < 0.05). The proportion of polypeptides with mass above 5 kDa in the other three bacteria was significantly reduced, which was consistent with the molecular weight distribution of wheat peptides prepared by enzymatic hydrolysis in a previous study (30). In general, the molecular weight distributions of peptides fermented by the **four** taxa were mostly concentrated at approximately 2 kDa, which shows that our fermentation was highly effective. Most proteins are hydrolyzed into peptides and amino acids. The reason for this difference may be that different taxa release different amounts and types of proteases during fermentation, thereby resulting in different lengths of polypeptide chains and different kinds of terminal amino acids (44).

**Table 2:** Molecular weight distribution of quinoa polypeptides hydrolyzed by different bacterial taxa.

**Sample**	**Molecular weight**			
	<**2 kDa**	**2–5 kDa**	**5–10 kDa**	>**10 kDa**
*Lactococcus Lactis subsp*	75.63%	15%	7.08%	2.3%
*Lactobacillus acidophilus*	82.58%	11.96%	4.27%	1.17%
*Lactobacillus paracasei*	83.36%	11.98%	3.91%	0.74%
*Lactobacillus fermentum*	82.58%	12.41%	4.14%	0.87%

In addition, the structure and amino acid sequence of peptides are closely related to their functions. Studies have shown that short peptides consisting of a small number of amino acids are more biologically active (45). The molecular weights of the peptides produced by the four bacteria in the figure were mainly distributed below 1 kDa, which leads to the assumption that at least some of the peptides produced by the fermentation of these four bacteria are likely to have a high degree of biological activity. This will be investigated in a subsequent study using methods such as UPLC-MS (32).

### 3.5. Amino acid profile

As shown in Table 3, among the non-essential amino acids obtained from the quinoa, Glu was the most abundant (*Lactococcus subsp*. 31%, *Lactobacillus acidophilus* 32%, *Lactobacillus paracasei* 32.8%, and *Lactobacillus fermentum* 34.6%), followed by Asp, Arg, Pro, Set, Gly, and Ala. Among the essential amino acids, Lys was the most abundant, while Met was the least. These findings are consistent with those reported by Daliria et al. (46). After fermentation by *Lactococcus subsp*. and *Lactobacillus paracasei*, the essential amino acid content increased, reaching nearly 40% of the total amino acid content. The hydrophobic amino acid content in the fermentation broth produced by *Lactococcus Lactis subsp*. fermentation was ~1.84 mg/mL, the hydrophobic amino acid content in the fermentation broth produced by *Lactobacillus acidophilus* fermentation was ~1.73 mg/mL, the hydrophobic amino acid content in the fermentation broth produced by *Lactobacillus paracasei* was ~1.9 mg/mL, and the content of hydrophobic amino acids in the fermentation broth produced by *Lactobacillus fermentum* was ~1.15 mg/mL; this was similar to the findings of Hong et al. (47). Fermentation by different microbial taxa yields different amino acid contents. This difference is partially explained by the different enzymes produced by different taxa during the fermentation process (48). Ultimately, different amino acid profiles and contents will result in products with different biological effects.

**Table 3:** Amino acid (mg/mL dry matter) content of proteins derived from quinoa.

	***Lactococcus Lactis subsp***	***Lactobacillus acidophilus***	***Lactobacillus paracasei***	***Lactobacillus fermentum***
**Non-essential amino acids (NEAA)**				
Asp	0.740243	0.677303	0.7644	0.4717
Glu	1.15912	1.09968	1.23499	0.816408
Ser	0.307253	0.228471	0.261846	0.152954
Gly	0.314376	0.214453	0.256537	0.167837
Arg	0.550059	0.5306	0.429055	0.314009
Ala	0.277728	0.260303	0.297276	0.180522
Pro	0.324337	0.341649	0.301068	0.256688
**Total non-essential amino acids (TNEAA)**				
	3.673116	3.352459	3.545172	2.360118
**Essential amino acids (EAA)**				
His	0.307683	0.159349	0.194357	0.102642
Thr	0.301064	0.177913	0.217237	0.112261
Tyr	0.125257	0.110893	0.131815	0.0981539
Cys	0.063697	0.0377218	0.0438721	0.033135
Val	0.288896	0.254439	0.285763	0.169513
Met	0.0517005	0.0550898	0.0587897	0.0300993
Phe	0.293717	0.2611	0.304678	0.161816
Ile	0.233744	0.218467	0.254272	0.132495
Leu	0.370706	0.33916	0.394822	0.214088
Lys	0.406337	0.392661	0.435372	0.269672
**Total essential amino acids (TEAA)**				
	2.4428015	2.0067936	2.3209778	1.3238752
Total amino acids (TAA)	6.1159175	5.3592526	5.866147	3.6839932

### 3.6. Stability studies

#### 3.6.1. Thermal stability

**Figure 6:**
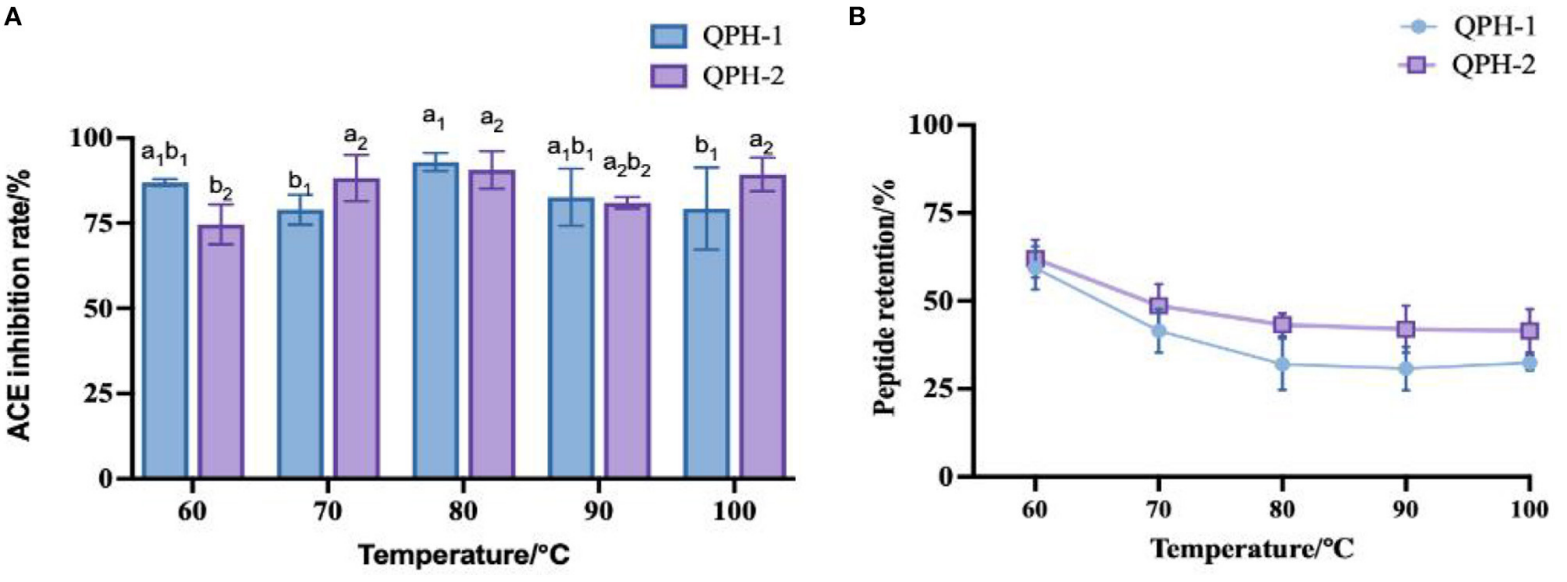
**(A)** Effect of samples treated with different temperatures on ACE inhibition rates. **(B)** Effect of samples treated with different temperatures on peptide yields. Different letters indicates the significant differences among samples (*p*-value ≤0.05).

Combined with the peptide retention rate, we can see that with increasing temperature, the overall peptide retention rate first decreased and then stabilized. As shown in Figure 6, at the temperature of 60°C, the retention rate of the QPH-1 peptide reached ~60%, while that of QPH-2 was ~65%. This result might be attributable to the fact that the molecular weight of QPH-1 is larger, and the molecular structure more complex, so the chance of irreversible denaturation by high-temperature decomposition is greater, thus rendering the retention rate of QPH-1 peptide to be generally smaller than that of QPH-2. The ACE inhibition rates of QPH-1 and QPH-2 was less sensitive to the temperature and the ACE activity could still be maintained at high temperature; this resembles the heat stability of Arthrospira platensis protein (49). This may be because ACE inhibitory peptides are low molecular weight peptides, such that heating cannot destroy their structure, so the effect on their function is minimal.

#### 3.6.2. pH stability

**Figure 7:**
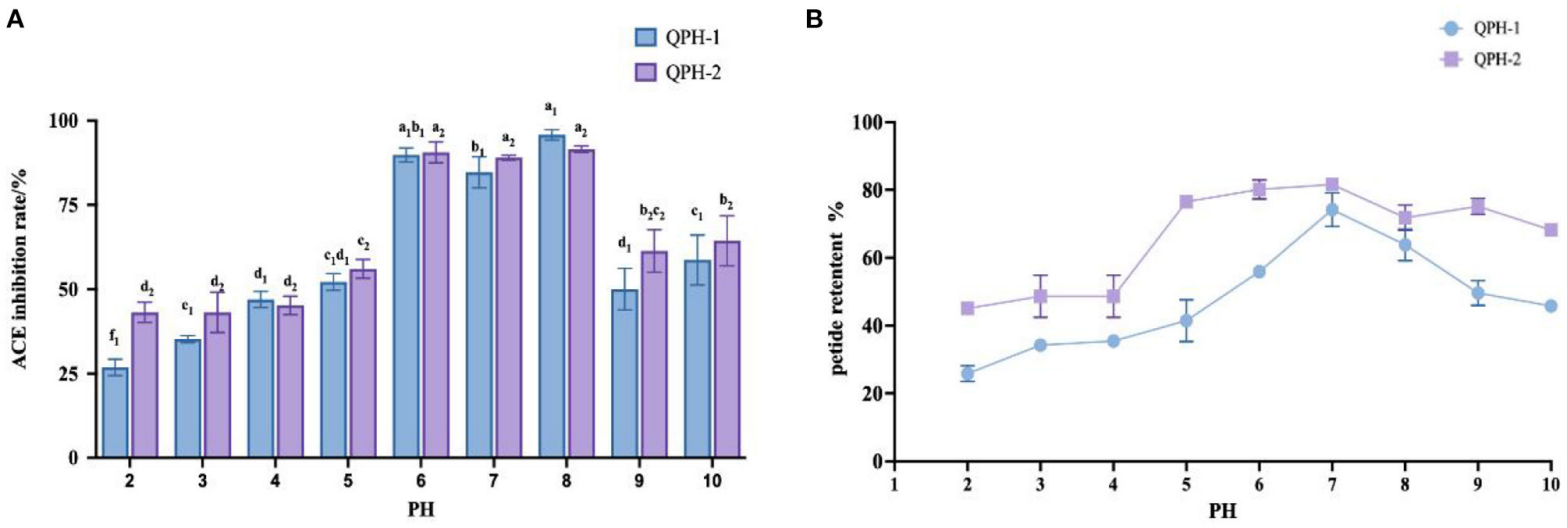
**(A)** Effect of samples treated with different pH on ACE inhibition rates. **(B)** Effect of samples treated with different pH on peptide yields. Different letters indicates the significant differences among samples (*p*-value ≤ 0.05).

As shown in Figure 7, the ACE inhibition and peptide retention rates of QPH-1 and QPH-2 were significantly different under different pH environments. In the strongly acidic environment, the ACE inhibition rate was significantly decreased (*p* < 0.05) and the ACE inhibition rate was maintained between 25 and 50% for QPH-1 and 40–50% for QPH-2; in the alkaline environment, the ACE inhibition rates of QPH-1 and QPH-2 were also significantly different than those in the acidic environment (*p* < 0.05); at pH = 6–8, there was no significant difference in the ACE inhibition of QPH-1 and QPH-2. This may be because racemic and deamidation reactions occur under strongly acidic or alkaline conditions, and the L-amino acids they contain are converted to D-amino acids, thereby forming a mixture of L-and D-amino acids and causing changes in the structural properties of the compounds, such as polarity and spatial location (28). It is also possible that under strongly acidic and alkaline conditions, the molecular structures of peptides become more ordered and compact, while hydrophobic amino acids are localized to the interior. The hydrophobic amino acids are often the site where some reactive groups of the protein perform their functions, so the functional activity is consequently reduced. Strongly acidic and alkaline conditions may cause some polar hydrophilic amino acids located on the surface of the protein to break from the polypeptide chain, which in turn causes a decrease in the retention of the polypeptide.

#### 3.6.3. Stability of metal ions

Food products are exposed to different metal containers during their processing and transportation, and the effects of different metal ions on the activity of quinoa hypotensive peptides (QPH-1 and QPH-2) differed significantly (*p* < 0.05) in Table 4. The effects of different ions on the retention of QPH-1 and QPH-2 peptides were ranked as Ca^2+^ >Zn^2+^ >K^+^ >Ba^2+^. The inhibition of ACE after the addition of Ca^2+^ changed to 75%. This may be because the addition of metal ions destroyed the structure of the peptides by breaking the chemical bonds existing between them, thereby resulting in the exposure of their hydrophobic groups, the reduction of their solubility, and other property changes. Therefore, during peptide food processing or transportation, measures should be taken to minimize their contact with vessels containing Ca^2+^ to maintain the peptide yield as well as hypotensive activity.

**Table 4:** Effects of different metal ions on the peptide yield and ACE inhibition rate.

**Metal ions**	**ACE inhibition (%)**		**Peptide retention (%)**	
	**QPH-1**	**QPH-2**	**QPH-1**	**QPH-2**
K^+^	55 ± 4.00^b^	55.33 ± 2.08^b^	87.98 ± 10.73^a^	91.56 ± 12.39^a^
Ca^2+^	75 ± 6.00^a^	76.67 ± 5.03^a^	95.14 ± 6.19^a^	91.56 ± 12.39^a^
Zn^2+^	58.67 ± 8.39^b^	56 ± 12.53^b^	98.71 ± 10.73^a^	98.71 ± 3.10^a^
Ba^2+^	42 ± 5.20^c^	45.33 ± 5.05^b^	95.14 ± 6.19^a^	95.14 ± 6.20^a^

Different letters indicates the significant differences among samples (*p*-value ≤ 0.05).

#### 3.6.4. Stability of organic solvents

The resistance of bioactive peptides to enzymes is important for allowing them to maintain their integrity and execute their biological functions, so this property is essential for peptides used as nutritional food ingredients As shown in Table 5, after pepsin and trypsin treatment, the peptide retention rate of both QPH-1 and QPH-2 decreased significantly. The ACE inhibition rate of QPH-1 also decreased significantly, unlike that of QPH-2, likely because QPH-2 has a smaller molecular weight and simple structure, such that following complete enzymatic cleavage at the enzymatic digestion site, the structure would not be destroyed during digestion, and the activity would hence be more stable, conferring QPH-2 a certain tolerance to the gastrointestinal tract. In contrast, the peptide chain length and structure of QPH-1 are more complex, so more hydrophobic groups would be susceptible to degradation by enzymatic reactions, and the arrangement order and structure of its constituent amino acids would be changed to ultimately reduce its activity. It is also possible that its activity would be reduced because it is more sensitive to environmental changes during the process of simulated gastrointestinal digestion, thus altering its structure.

**Table 5:** Effect of pepsin and trypsin on peptide yield and ACE inhibition.

**Protease species**	**ACE inhibition (%)**		**Peptide retention (%)**	
	**QPH-1**	**QPH-2**	**QPH-1**	**QPH-2**
Pepsin	78 ± 2.6	83.33 ± 2.1	82.67 ± 1.9	86.4 ± 1.0
Trypsin	77.34 ± 2.1	81.67 ± 1.5	78.2 ± 1.6	83.57 ± 2.1

ACE inhibitory peptides can be divided into 3 categories according to the stability of gastrointestinal digestion. The first is the true inhibitory type, whereby after conducting an *in vitro* simulation experiment, the inhibitory activity of ACE polypeptide has no effect; the second is the substrate type, whereby after conducting an *in vitro* simulation experiment, the inhibitory activity of ACE polypeptide decreased significantly; the third is the precursor type, whereby after conducting an *in vitro* simulation experiment, the inhibitory activity of ACE polypeptide increased significantly. Therefore, quinoa peptide belongs to the substrate type.

#### 3.6.5. Stability of organic solvents

**Figure 8:**
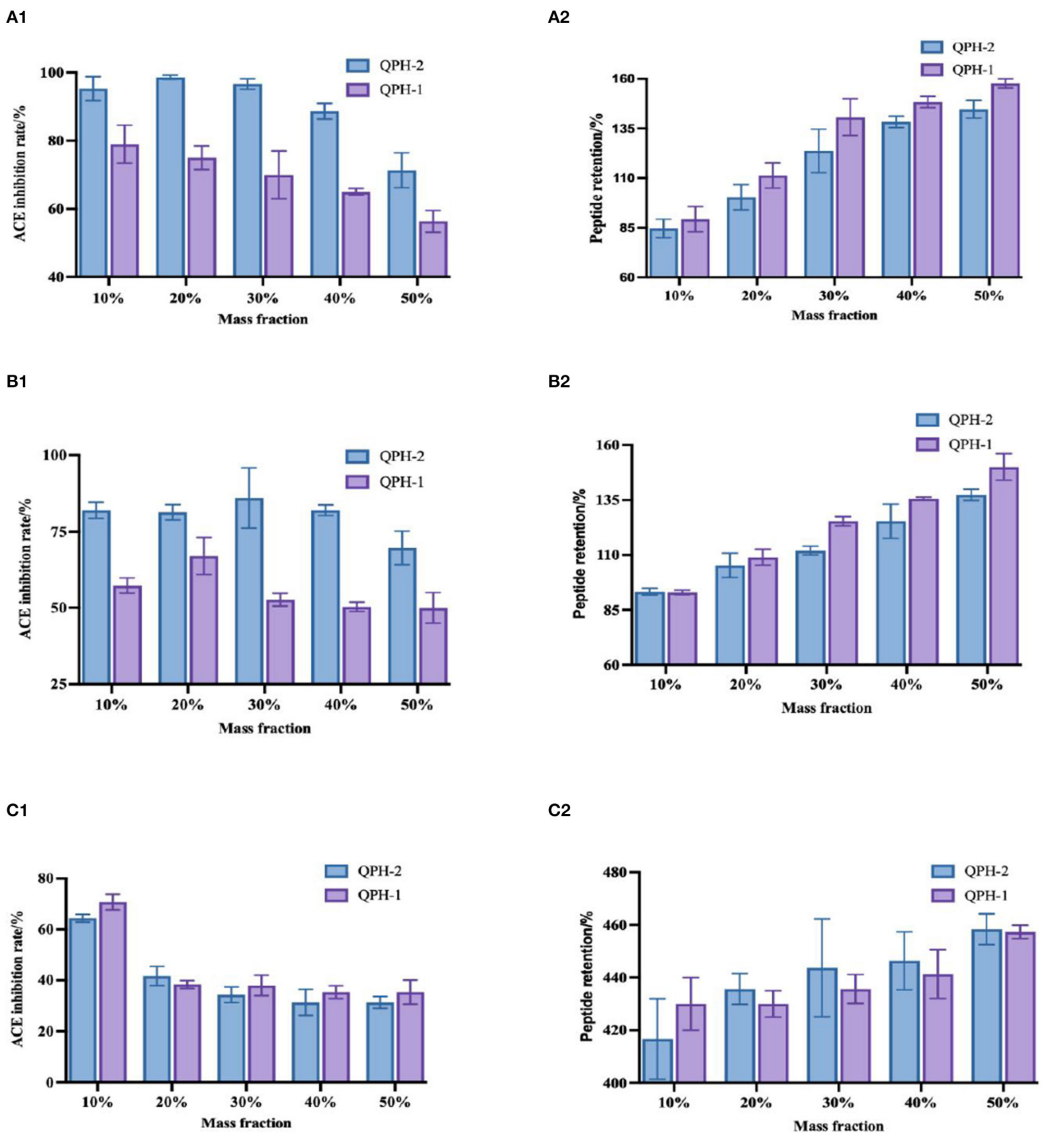
**(A1–C1)** Shows the changes in ACE inhibition in the samples treated with different mass fractions of methanol, ethanol, and propanol, respectively. **(A2–C2)** Show the changes in peptide retention in the samples treated with different mass concentrations of methanol, ethanol, and propanol, respectively.

As shown in Figure 8, the three organic solvents all increased the retention of the quinoa peptides, with a significant difference observed with increasing mass fraction of the organic solvents. On the one hand, organic solvents lowered the dielectric constant of the aqueous solution, increased the Coulomb gravitational force between the charges of solute molecules, and reduced the protein solubility. In addition, they also compressed the surface splash layer of the protein molecules by acting with the solvent water, such that the proteins dehydrated and aggregated, while the protein content measured by the TCA method mainly measured the number of peptide bonds, so organic solvents would hinder the decomposition of proteins and further increase the peptide content. As proteins dehydrate and aggregate, the hydrophobic and aromatic amino acids with functional activity are wrapped by hydrophilic amino acids, which leads to a decrease in ACE activity. As the mass fraction of organic solvent increases, they become wrapped more tightly, thereby decreasing the ACE inhibition rate.

#### 3.6.6. Stability of light

**Figure 9:**
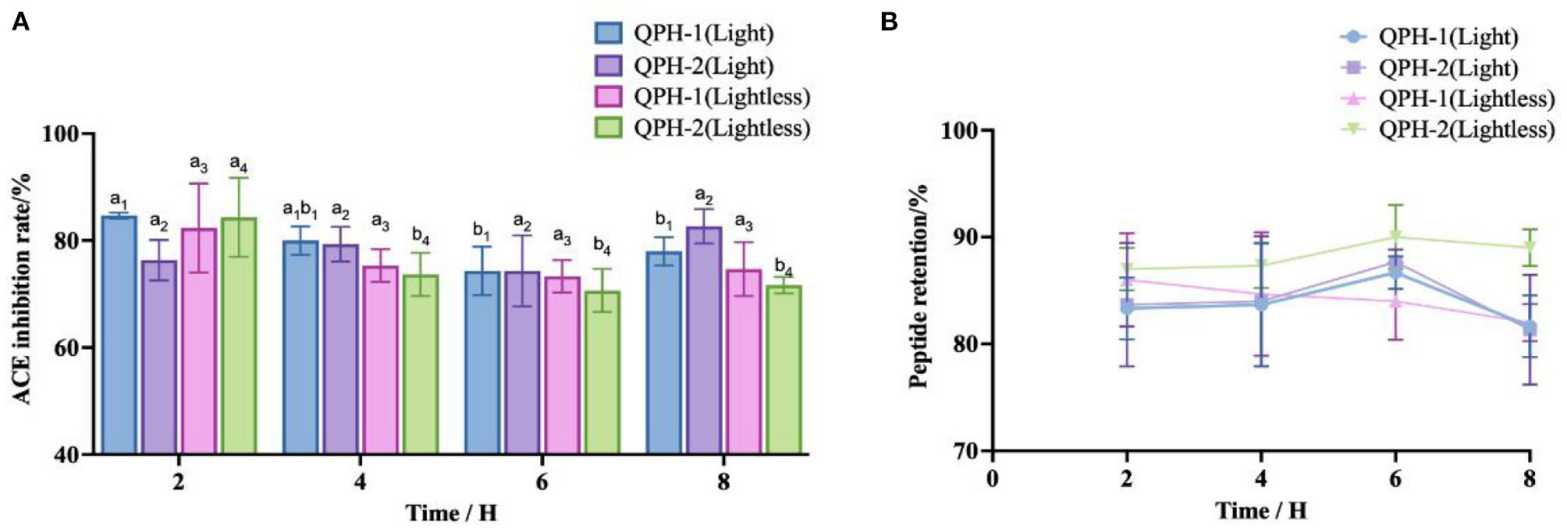
**(A)** ACE inhibition of samples treated with different light conditions and lengths of time. **(B)** Peptide retention of samples treated with different light conditions and different lengths of time. Different letters indicates the significant differences among samples (*p*-value ≤ 0.05).

From the Figure 9, we can see that with the availability of light conditions and the extension of time, the polypeptide retention rates of QPH-1 and QPH-2 were essentially maintained at 80–90%. The ACE inhibitory activity of QPH-1 (light) decreased significantly after 6 h of illumination (*p* < 0.05). Within 2–8 h, there was no significant difference in ACE inhibitory activity between QPH-2 (light) and QPH-1 (lightless) (*p* > 0.05). After 2 h, the ACE inhibitory activity of QPH-2 (lightless) decreased significantly (*p* < 0.05). These results indicate that during the storage of peptides, light would exert little effect on their stability.

#### 3.6.7. Stability of measured ionic strength

**Figure 10:**
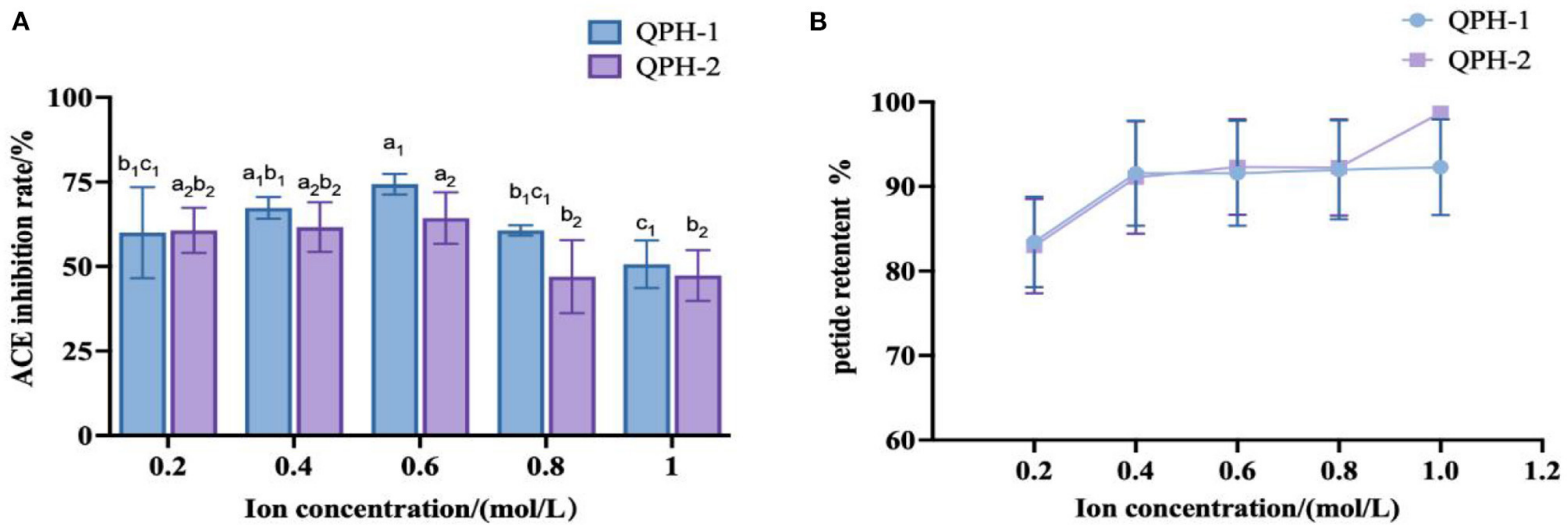
**(A)** ACE inhibition of samples treated with different ion concentrations. **(B)** Peptide retention of samples treated with different ion concentrations. Different letters indicates the significant differences among samples (*p*-value ≤ 0.05).

As shown in Figure 10, the addition of different concentrations of NaCl did not greatly affect the retention of quinoa peptides. When the concentration of NaCl was 0.2–0.4 mol/L, the inhibitory activities of ACE in QPH-1 and QPH-2 were relatively stable; when the concentration of NaCl was 0.6 mol/L, the inhibitory activities of ACE in QPH-1 and QPH-2 increased slightly, and the concentration of NaCl was higher than 0.4 mol/L. When the concentration exceeded 0.6 mol/L, the inhibition rate of ACE of QPH-1 and QPH-2 decreased significantly, and the concentration of NaCl exhibited a negative correlation with the inhibition rate of ACE. This result may be because Cl^−^ readily accepts hydrogen bonds, and this property can break the hydrogen bonds existing between protein molecules to a large extent to lead to their decomposition (50). A certain amount of NaCl had little effect on the ACE inhibitory activity of quinoa hypotensive peptides, and when the concentration of NaCl was high, it markedly affected the ACE inhibitory activity, therefore, desalination must be performed.

### 3.7. Results of antibacterial activity

**Figure 11:**
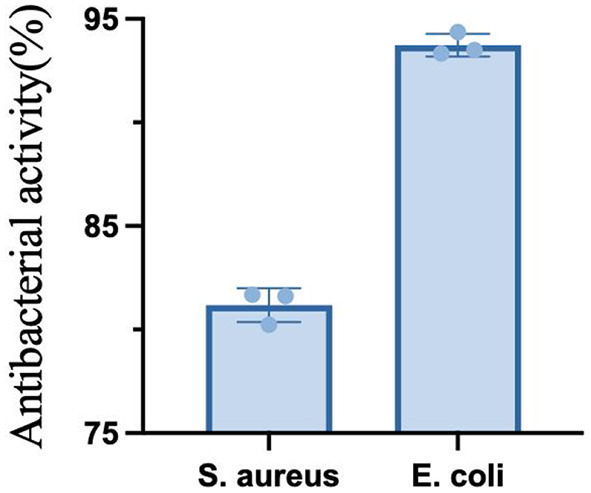
Antimicrobial activity of quinoa peptides.

As shown in the Figure 11, the fermented quinoa peptides showed some inhibitory effect on both E. coli and S. aureus, and the inhibitory activity against E. coli was higher than that of S. aureus.

## 4. Conclusions

In this study, QF was first hydrolyzed with amylase and protease enzymes before being fermented with 16 different bacterial taxa for 40 h. Eight taxa that exhibited higher peptide production capacities were screened from the 16 taxa using peptide content as an indicator. From these 8 taxa, 4 taxa that produced peptides with smaller molecular weights were further screened by SDS-PAGE. The molecular weight distributions and amino acid contents of the peptides in the fermentation broths generated by the four taxa were analyzed by high-performance liquid chromatography. It was found that the molecular weights of the polypeptide in the fermentation broths produced by the four taxa were concentrated below 2 kDa. The majority (83.36%) of peptides in the fermentation broth produced by Lactobacillus paracasei had molecular weights <2 kDa after fermentation, the content of essential amino acids was higher, with the highest content of hydrophobic amino acids (~1.9 mg/mL)among the bacterial taxa tested, which was of great importance for the subsequent study of the peptide efficacy. Therefore, among 16 taxa, *Lactobacillus paracasei* was selected as the dominant taxon for the preparation of quinoa peptides by combining the results obtained for peptide yield, SDS-PAGE, peptide molecular weight distribution, and amino acid analysis. Through consulting the available literature, we found that the ACE inhibition rate and peptide retention rate of peptides with molecular weights between 0 and 3 kDa tend to be relatively stable under unstable environmental conditions. This study provides a useful reference for the development and production of fermented products containing a high content of peptides. At the same time, the inhibitory activity of quinoa peptide after fermentation on E. coli was obvious. Subsequent studies may focus on the elaboration the blood pressure lowering effect of quinoa peptide, identification of efficacy peptides, and the use of cellular models to reveal the activity of efficacy peptides derived from quinoa.

## Data availability statement

The original contributions presented in the study are included in the article/supplementary material, further inquiries can be directed to the corresponding authors.

## Author contributions

LW: conceptualization and supervision. XF: data curation and writing—original draft preparation. XLiu: visualization and investigation. GP, RA, XLi, XW, AA, and RM: resources. CQ: software and validation. XM: writing—reviewing and editing. JY: validation. All authors have read and agreed to the published version of the manuscript.
